# When intact mucosa hides a malignancy: solid-type primary intraosseous carcinoma (PIOC, NOS) of the mandible: a case report and literature review

**DOI:** 10.3389/fdmed.2026.1918737

**Published:** 2026-10-14

**Authors:** Bassel Tarakji, Maya Al-Jokhadar, Wafaa Talas, Qusay Alshblak, Faisal S. Alhedyan, Mohammed A. S. Abuelqomsan, Faisal Mehsen Alali, Deema Alshammery, Ali Alrafedah, Mohammed Alshammari, Mohammad Zakaria Nassani, Anas Alsalhani

**Affiliations:** 1 Department of Oral and Maxillofacial Surgery and Diagnostic Sciences, College of Dentistry, Prince Sattam Bin Abdulaziz University, Al-Kharj, Saudi Arabia; 2 Department of Oral Histology and Pathology, College of Dentistry, Arab International University, Damascus, Syria; 3 Department of Oral and Maxillofacial Surgery, Ibn Al-Nafees Hospital, Ministry of Health, Damascus, Syria; 4 Department of Oral and Maxillofacial Surgery, Ibn Al-Nafees Hospital, Ministry of Health, Damascus Health Directorate, Damascus, Syria; 5 Department of Conservative Dentistry, College of Dentistry, Prince Sattam Bin Abdulaziz University, Al-Kharj, Saudi Arabia; 6 Department of Preventive Dentistry, College of Medicine and Dentistry, Riyadh Elm University, Riyadh, Saudi Arabia; 7 Department of Restorative and Prosthetic Dental Sciences, College of Dentistry, Dar Al Uloom University, Riyadh, Saudi Arabia; 8 Department of Removable Prosthodontics, Faculty of Dentistry, University of Aleppo, Aleppo, Syria; 9 Department of Dentistry, Vision Colleges, Riyadh, Saudi Arabia; 10 Department of Histology and Pathology, Faculty of Dentistry, Hama University, Hama, Syria

**Keywords:** CARE guidelines, case report, histopathology, keratin pearl, mandible, odontogenic carcinoma, PIOC NOS, primary intraosseous carcinoma

## Abstract

**Introduction:**

Primary intraosseous carcinoma, not otherwise specified (PIOC, NOS) is a rare odontogenic carcinoma that arises within the jawbone from odontogenic epithelial remnants, with no initial connection to the oral mucosa. Its non-specific clinical and radiographic features overlap with benign odontogenic and inflammatory lesions, so diagnosis is often delayed and rests on histopathology. The 2017 and 2022 World Health Organization (WHO) classifications recognize PIOC, NOS without formal subtypes; earlier schemes described a solid (*de novo*) type arising without a cystic or tumorous precursor.

**Case:**

A 53-year-old man with no tobacco or alcohol history presented with painful right mandibular and cheek swelling, purulent discharge through a cutaneous fistula and mobile right mandibular molars, persisting despite management as an odontogenic abscess. The oral mucosa was intact and there was no sensory disturbance. Cone-beam computed tomography showed a destructive radiolucent lesion from the right mandibular first molar to an impacted third molar, with buccolingual expansion, cortical thinning and probable lingual perforation. After extraction of the molars, incisional biopsy showed invasive well-differentiated keratinizing squamous cell carcinoma with keratin pearls and bone trabeculae encased by tumor nests, without a cystic or odontogenic tumor component. Staging CT identified no suspicious primary tumor within the imaged regions. The diagnosis was PIOC, NOS under WHO 2022; the tumor was well differentiated (Grade I) and corresponded to the historically described solid (*de novo*) type. Segmental mandibulectomy with right neck dissection revealed a 7-cm tumor with perineural and lymphovascular invasion and four positive level I lymph nodes; margins were clear. Postoperative radiotherapy (40 Gy in 20 fractions) with concurrent cisplatin followed. In August 2026, CT showed an operative-bed mass suspicious for locoregional recurrence, and paclitaxel–carboplatin chemotherapy began in September 2026.

**Conclusion:**

Solid-type PIOC, NOS can mimic benign odontogenic and inflammatory lesions beneath an intact mucosa. A destructive jaw lesion that fails to respond to treatment for infection should be biopsied without delay. Keratin pearls, infiltrative epithelial nests and bone invasion without mucosal continuity are decisive for diagnosis, and the nodal metastasis and suspected recurrence in this case underline the need for continued oncologic surveillance.

## 1 Introduction

Primary intraosseous carcinoma, not otherwise specified (PIOC, NOS) is a rare, aggressive odontogenic malignancy that originates within the jawbone from epithelial remnants of odontogenic origin such as the rests of Malassez, the dental lamina, or the lining epithelium of odontogenic cysts without any initial connection to the oral mucosa (1, 2). The lesion was first described by Loos in 1913 as a central squamous cell carcinoma of the jaw and was subsequently incorporated into World Health Organization (WHO) classifications of odontogenic carcinomas (1, 3, 4).

PIOC accounts for a small fraction of all oral malignancies and predominantly affects middle-aged to older male patients, with a marked predilection for the posterior mandible that reflects the regional distribution of odontogenic epithelium (3, 5). In the most comprehensive systematic review to date, which pooled 257 cases from 109 published reports, the mandible was affected approximately seven times more often than the maxilla (7:1), the posterior region was the commonest site, and the mean age at diagnosis was 57.3 years. A male predilection was evident, with men accounting for 69.3% of cases, corresponding to a male-to-female ratio of approximately 2.3:1 (5). Historically described pathways include *de novo* development from odontogenic epithelial rests and malignant transformation of pre-existing odontogenic cysts, such as residual, radicular or dentigerous cysts (2, 6, 7).

Clinically, PIOC often presents as a slowly enlarging intraosseous mass associated with swelling, tooth mobility or mild pain. In some patients the lesion remains asymptomatic for extended periods, mimicking benign jaw pathologies such as odontogenic cysts, chronic osteomyelitis, or the odontogenic keratocyst (8, 9). Because of these non-specific radiographic and clinical features, PIOC is frequently misdiagnosed initially, which may delay treatment and adversely influence prognosis (9, 10).

Histopathologically, PIOC is characterized by nests and islands of well-differentiated squamous epithelial cells displaying keratin pearl formation, cellular pleomorphism and bone invasion within a fibrous stroma (9, 11). The absence of a connection to the oral mucosa, together with unequivocal bone involvement, is critical to the diagnosis. Diagnostic confirmation rests on the criteria proposed by Suei et al., which require absence of an ulcer of the overlying oral mucosa, absence of another primary tumor at the time of diagnosis and during at least 6 months of follow-up, and histological evidence of squamous cell carcinoma (12).

Despite advances in imaging and pathology, the prognosis of PIOC remains guarded. Several clinical series have reported five-year overall survival of approximately 36%–39%, with histological grade and nodal disease among the prognostically important factors identified across studies (9, 13, 14). Owing to its rarity and to overlapping features with other intraosseous lesions, early biopsy and histopathological assessment remain essential for accurate diagnosis and prompt management (10, 15).

This report presents a clinically and histologically confirmed case of solid-type PIOC, NOS of the mandible and reviews the relevant literature. Because the clinical and radiographic features of this tumor are non-specific and are shared with benign odontogenic and inflammatory jaw lesions, imaging can raise suspicion of an aggressive central process but cannot establish the diagnosis. The emphasis of this report is therefore on maintaining a low threshold for early incisional biopsy when a destructive intraosseous lesion behaves aggressively, and on the microscopic features on which definitive recognition ultimately depends, so that diagnostic delay is avoided and patient outcomes are optimized.

## 2 Case presentation

### 2.1 Patient information

A 53-year-old male was referred for evaluation of a radiolucent lesion of the right posterior mandible. He presented with painful swelling of the right mandibular region and cheek, associated with purulent discharge through a cutaneous fistula. The condition was initially considered an odontogenic abscess and was treated with antibiotics and local drainage. However, the swelling, pain, and purulent discharge persisted. Following radiographic evaluation (Section 2.4.1), the right mandibular first and third molars, which were located within the affected region, were extracted. Abnormal, friable-appearing tissue was encountered in the affected area after extraction, prompting histopathological investigation. An incisional biopsy was subsequently obtained from the abnormal tissue.

The medical history was unremarkable. He had never used tobacco in any form and denied alcohol consumption, and there was no history of a previous malignancy or of head-and-neck radiotherapy — negatives of particular relevance to the exclusion of metastatic and surface-derived disease. There was no relevant family history of head-and-neck malignancy or hereditary cancer syndromes. Written informed consent for publication, including the use of clinical and radiographic images in anonymized form, was obtained prior to manuscript preparation.

### 2.2 Clinical findings

Extra-oral examination was notable for a cutaneous fistula of the right cheek, at the site that had been managed conservatively one month earlier. There was no trismus and no sensory deficit in the distribution of the inferior alveolar or mental nerve.

Intra-oral examination revealed mobility of the involved right mandibular molar teeth. The overlying oral mucosa was entirely intact, with no ulceration, no surface growth and no intra-oral sinus tract. Tooth mobility occurring in the absence of any mucosal ulceration, surface lesion or intra-oral sinus tract that might account for it was regarded as clinically discordant and prompted radiographic evaluation of the underlying bone.

### 2.3 Timeline

A cutaneous fistula of the right cheek was first noted and managed approximately one month before diagnosis, in the context of painful swelling and purulent discharge initially managed as an odontogenic abscess. Following persistence of symptoms, CBCT was performed on 30 August 2025. The involved mandibular molars were then extracted, and the abnormal tissue encountered at the extraction sites prompted an incisional biopsy on 7 September 2025, with a histopathological diagnosis of PIOC, NOS under WHO 2022 on 10 September 2025; the tumor was well differentiated (Grade I) and corresponded to the historically described solid (*de novo*) type. Subsequent management included staging evaluation, definitive oncologic surgery, postoperative chemoradiotherapy, and continued oncologic follow-up. In August 2026, follow-up imaging demonstrated findings suspicious for locoregional recurrence, followed by systemic chemotherapy in September 2026. A complete CARE-format chronological summary is provided in Supplementary Table S1.

### 2.4 Diagnostic assessment

#### 2.4.1 Imaging findings

CBCT and panoramic imaging revealed a radiolucent intraosseous lesion involving the right posterior mandible, extending from the first molar to the region of the impacted third molar (Figures 1A–C). The sagittal CBCT view demonstrated an intraosseous radiolucent lesion with irregular bone resorption and thinning of the inferior cortical border (Figure 1A). The axial section showed buccolingual expansion of the mandibular body with marked cortical thinning and probable focal cortical discontinuity or perforation along the lingual aspect; the impacted third molar was displaced inferiorly toward the mandibular canal (Figure 1B). The lingual cortical defect may account for the associated cutaneous fistula and suggests limited extension into the adjacent soft tissues. The panoramic radiograph displayed a well-defined radiolucent area in the right posterior mandible with partial alveolar bone loss and loss of the trabecular pattern (Figure 1C). Collectively, these findings supported a central (intraosseous) origin of the lesion and indicated aggressive behavior.

**Figure 1:**
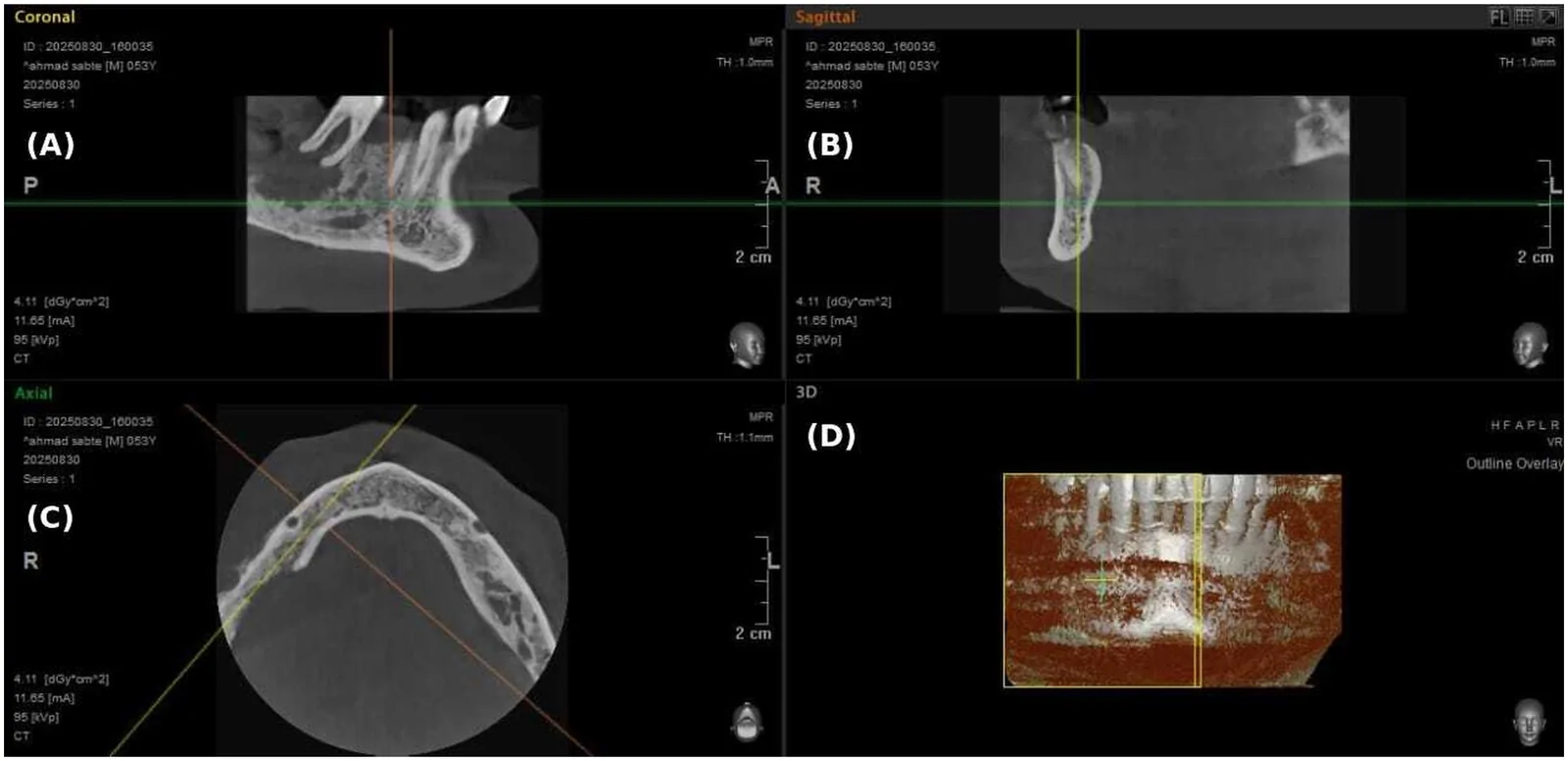
Multiplanar cone-beam computed tomography (CBCT) evaluation of the right mandibular lesion. **(A)** Coronal reconstruction demonstrates an intraosseous abnormality in the right posterior mandible, with disruption of the normal trabecular architecture. **(B)** Sagittal reconstruction shows the anteroposterior location of the involved region. **(C)** Axial reconstruction confirms the intraosseous epicenter and demonstrates its relationship to the buccal and lingual cortices. **(D)** Three-dimensional volume-rendered reconstruction shows the lesion's anatomic location relative to the dentition. Together, the multiplanar views demonstrate the intraosseous epicenter of the lesion.

Subsequent staging contrast-enhanced CT of the brain, neck, and chest, performed on 17 September 2025, was used to assess disease extent and to search for a potential primary tumor elsewhere. No suspicious primary tumor was identified within the imaged regions.

#### 2.4.2 Diagnostic procedure

Following extraction of the involved mandibular molars, abnormal, friable-appearing tissue was encountered within the intraosseous lesion. Under local anesthesia and using an intra-oral approach, an incisional biopsy was performed through the extraction sockets. Representative tissue was obtained from within the intraosseous compartment, where the tumor had resorbed the trabecular bone and formed a soft-tissue mass occupying the medullary cavity. The specimen comprised tumor tissue together with fragments of residual trabecular bone. Tissue was submitted for histopathological examination in 10% neutral-buffered formalin.

No intra-operative complications were encountered and the patient tolerated the procedure well. Immunohistochemical and molecular ancillary studies were not performed; this limitation is addressed transparently in Section 3.3.

#### 2.4.3 Microscopic findings

Histopathological examination of the incisional biopsy revealed invasive islands and cords of well-differentiated squamous epithelial cells within a fibrous connective-tissue stroma, with no continuity with surface epithelium (Figure 2A). The tumor showed prominent squamous differentiation and keratinization within the invasive epithelial islands (Figure 2B). Nuclear pleomorphism, individual-cell keratinization, and prominent keratin pearl formation were evident at higher power (Figure 2C). Mitotic activity was also identified histologically. Bone trabeculae partially encased by malignant epithelial islands were also identified, confirming intraosseous invasion (Figure 2D). No evidence of a pre-existing odontogenic cystic lining or odontogenic tumor component was identified.

**Figure 2:**
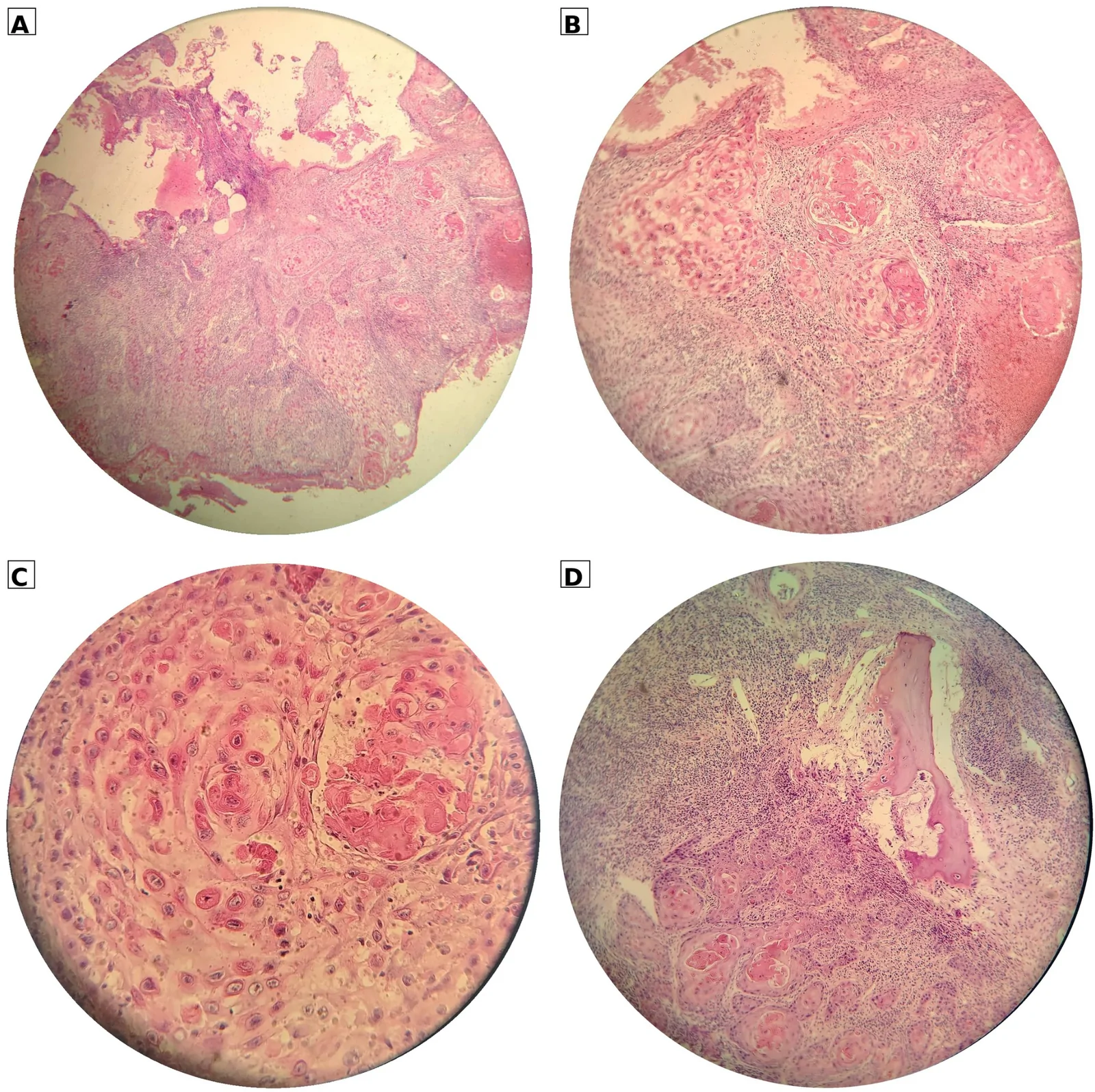
Histopathological features of the initial incisional biopsy (hematoxylin and eosin; original magnification: A, ×4; B, ×10; C, ×40; D, ×10). **(A)** Low-power view showing invasive nests and islands of well-differentiated squamous cell carcinoma within connective tissue, with an associated inflammatory stromal response; no continuity with surface epithelium is present. **(B)** Intermediate-power view demonstrating invasive well-differentiated keratinizing squamous cell carcinoma with prominent squamous differentiation and keratinization. **(C)** High-power view showing nuclear pleomorphism, individual-cell keratinization, and prominent keratin pearl formation. **(D)** Tumor–bone interface showing a bone fragment closely apposed to and surrounded by malignant squamous epithelial nests, with associated stromal inflammation.

#### 2.4.4 Diagnostic challenges and differential diagnosis

The pre-operative working differential diagnosis for a destructive radiolucent posterior mandibular lesion with an intact mucosa included odontogenic cyst (radicular, residual or dentigerous); odontogenic keratocyst; ameloblastoma; chronic suppurative osteomyelitis; metastatic carcinoma to the jaw; intraosseous mucoepidermoid carcinoma; ameloblastic carcinoma; and, given the cortical destruction, cutaneous fistula and aggressive radiographic appearance, primary intraosseous carcinoma.

None of these entities could be excluded on clinical and radiographic grounds alone. The initial painful swelling, purulent discharge and cutaneous fistula had led to a provisional diagnosis of an odontogenic abscess and treatment with antibiotics and local drainage; persistence of the symptoms despite this treatment contributed to further investigation. A destructive radiolucent posterior mandibular lesion presenting with tooth mobility and a cutaneous fistula, but without mucosal ulceration, can readily be attributed to a radicular or dentigerous cyst, an odontogenic keratocyst, chronic osteomyelitis or an ameloblastoma (8, 9, 15). The intact oral mucosa, simultaneously the defining feature of PIOC and a principal reason it can evade early recognition, removed the finding that might otherwise have directed earlier attention to a malignant process. The cortical perforation evident on axial CBCT plausibly explains the cutaneous fistula and underscores that aggressive radiographic behavior should prompt biopsy rather than expectant management. An incisional biopsy was therefore considered essential for definitive characterization (2, 9, 15). A focused comparison of the principal differential diagnoses is provided in Table 1.

**Table 1:** Principal clinicopathological differential diagnoses for a destructive radiolucent posterior mandibular lesion with intact oral mucosa.

Entity	Typical features	Key histologic picture	Differentiating points from the present case	Ref.
Solid-type PIOC, NOS	Posterior mandible; middle-aged males; radiolucency with cortical destruction; intact mucosa	Infiltrative squamous islands; keratin pearls; bone invasion; no mucosal continuity; no precursor cyst or tumor	Final diagnosis in this case	(2, 4, 5)
Radicular/residual/dentigerous cyst	Well-defined corticated radiolucency, often around a non-vital tooth or impacted crown	Cystic epithelial lining without atypia; no invasion	Excluded: no cystic lining; invasive malignant epithelium present	(6, 7)
Odontogenic keratocyst	Mandibular body or ramus; corticated radiolucency; may behave aggressively	Parakeratinized corrugated lining; palisaded basal layer; no invasion	Excluded: no keratocystic lining; frank stromal invasion	(15, 18, 21)
Ameloblastoma	Posterior mandible; multilocular expansile radiolucency	Ameloblastic epithelium with reverse polarity and stellate reticulum; no keratin pearls or atypia	Excluded: squamous islands with keratin pearls and atypia, not ameloblastic	(3, 4)
Chronic suppurative osteomyelitis	Ill-defined radiolucency or sclerosis; may produce a fistula	Inflamed granulation tissue and necrotic bone; no malignant epithelium	Excluded: malignant epithelial islands invading bone	(8, 9)
Metastatic carcinoma to the jaw	Older patients; ill-defined radiolucency; known primary	Histology reflects the distant primary	Not supported: no previous malignancy or clinically apparent primary tumor, and staging CT identified no suspicious primary tumor within the imaged regions	(2, 12)
Intraosseous mucoepidermoid carcinoma	Posterior mandible; unilocular or multilocular radiolucency	Mucous, intermediate and epidermoid cells; mucin positive	Excluded: pure keratinizing squamous differentiation, no mucous cells	(3, 4)
Ameloblastic carcinoma	Aggressive radiolucency; rapid growth	Ameloblastic features with overt malignant cytology	Excluded: no ameloblastic differentiation	(4, 19)
Surface (alveolar) oral squamous cell carcinoma (OSCC) invading bone	Mucosal ulcer or mass with secondary bone invasion	Continuity with ulcerated surface mucosa	Excluded: overlying mucosa intact, no surface continuity	(2, 11)

Final discrimination from surface-derived and metastatic carcinoma requires confirmation of an intact overlying mucosa, exclusion of a distant primary, and histological evidence of carcinoma arising within bone.

#### 2.4.5 Final pathological diagnosis

The microscopic findings supported the diagnosis of primary intraosseous carcinoma, not otherwise specified (PIOC, NOS), under the 2022 WHO Classification of Head and Neck Tumors (3, 4); the tumor was well differentiated (Grade I). Because no cystic lining or odontogenic tumor component was identified, the tumor corresponds to the solid (*de novo*) type described in earlier classifications (2). The primary intraosseous origin was further supported by the intraosseous location of the biopsy, the central location of the lesion on imaging, and the intact overlying oral mucosa, with no ulceration or surface growth. Clinical evaluation revealed no history of previous malignancy, no systemic symptoms suggestive of an underlying malignancy, and no clinically apparent primary tumor elsewhere. Staging contrast-enhanced CT of the brain, neck, and chest performed on 17 September 2025 identified no suspicious primary tumor within the imaged regions. Together with clinical and radiologic follow-up extending beyond 6 months without documentation of an alternative primary tumor, these findings satisfied the diagnostic criteria of Suei et al. for primary intraosseous carcinoma (12).

### 2.5 Therapeutic intervention

Following histopathological confirmation, the patient was referred for definitive oncologic management. On 1 October 2025, he underwent segmental mandibulectomy of the right mandible with right neck dissection. Final surgical pathology, reported after decalcification of the bony resection specimen, demonstrated a 7-cm invasive well-differentiated keratinizing squamous cell carcinoma involving the gingiva and gingivobuccal fold and invading the underlying soft tissue, mandibular bone, and overlying skin, with fistula-tract formation. Perineural and lymphovascular invasion were identified. Four level I lymph nodes, submitted together with the attached submandibular gland, showed metastatic tumor, whereas the remaining 12 lymph nodes of the right neck dissection specimen were reactive and free of tumor (overall, 4 of 16 lymph nodes involved). All surgical margins were free of tumor. Postoperative radiotherapy commenced on 9 December 2025 and was delivered at 2 Gy per fraction for 20 fractions, for a total dose of 40 Gy; the course was extended by treatment interruptions. Concurrent cisplatin was administered during the radiotherapy course, including a documented dose of 50 mg on 14 January 2026.

### 2.6 Follow-up and outcomes

Postoperative contrast-enhanced CT of the head and neck performed on 30 October 2025 demonstrated postoperative changes in the right mandibular surgical bed. During subsequent oncologic follow-up, contrast-enhanced CT of the head, neck, and chest performed on 12 August 2026 demonstrated a new heterogeneous enhancing soft-tissue mass centered in the right mandibular operative bed, measuring approximately 4.5 × 3.9 × 4.0 cm and radiologically suspicious for locoregional recurrence (Figures 3A,B). A comparative radiologic assessment on 27 August 2026, using the postoperative CT of 30 October 2025 for comparison, confirmed the new irregular heterogeneous mass in the surgical bed and recommended clinical and histopathological correlation. In the absence of available histopathological confirmation of recurrence, the finding was considered a radiologically suspected locoregional recurrence. Systemic chemotherapy with paclitaxel (250 mg) and carboplatin (450 mg) was initiated on 21 September 2026.

**Figure 3:**
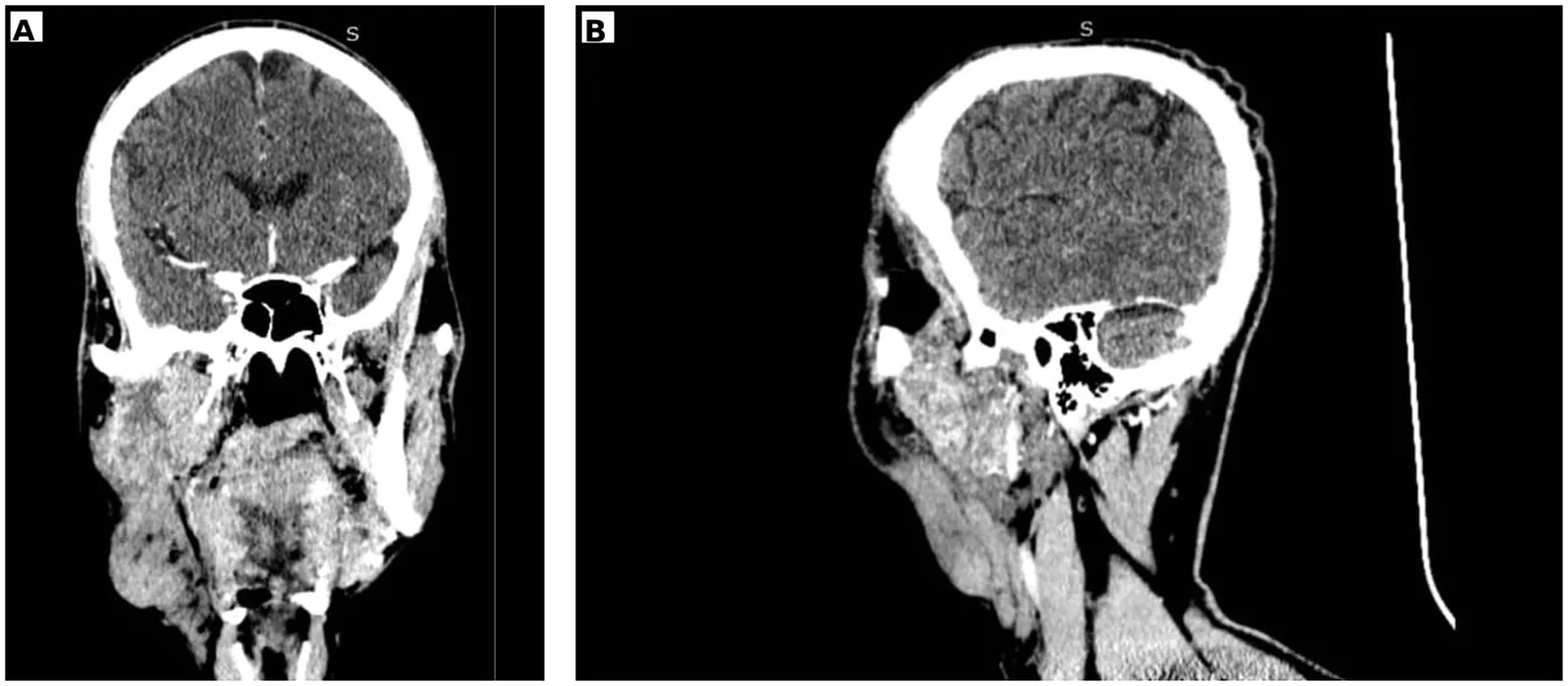
Follow-up contrast-enhanced computed tomography of 12 August 2026. **(A)** Coronal and **(B)** sagittal images demonstrate a new heterogeneous enhancing soft-tissue mass centered in the right mandibular operative bed, radiologically suspicious for locoregional recurrence.

## 3 Discussion

The findings in the present case can be compared directly with the published literature on PIOC, NOS.

### 3.1 Comparison with the literature

The published literature consistently indicates that PIOC, NOS is a rare odontogenic carcinoma with marked diagnostic complexity. In the systematic review by de Morais et al., primary intraosseous squamous cell carcinoma of the jaws most frequently involved the mandible, particularly the posterior region, and commonly affected middle-aged to older adults (5). These demographic and anatomical tendencies are consistent with the present case, a 53-year-old male with a posterior mandibular lesion.

In the series of Bodner et al., Huang et al. and Naruse et al., none of the reported clinical or radiographic findings swelling, tooth mobility, pain, paresthesia, radiolucency, cortical destruction or fistula formation proved pathognomonic (7–9); the present case reproduces that pattern, combining tooth mobility, a radiolucent mandibular defect, cortical thinning with probable perforation and a cutaneous fistula while the oral mucosa remained intact.

From a classification perspective, the WHO 2017 (4th) and 2022 (5th) classifications recognize PIOC, NOS as a single entity within the odontogenic carcinomas and no longer subdivide it formally (3, 4). Earlier schemes, including the classification of Waldron and Mustoe and descriptions summarized in subsequent WHO updates, distinguished carcinoma arising *de novo* (solid type), carcinoma arising from an odontogenic cyst, and carcinoma arising in association with an odontogenic tumor (2–4). Although no longer part of the formal WHO classification, this distinction by presumed developmental pathway remains descriptively useful. The solid (*de novo*) type is particularly uncommon because no pre-existing cystic lining or odontogenic tumor component is identified. This point is central to the present case, in which histopathological examination disclosed no precursor cyst or tumor, consistent with a solid (*de novo*) PIOC, NOS. The historically described developmental pathways are summarized schematically in Figure 4.

**Figure 4:**
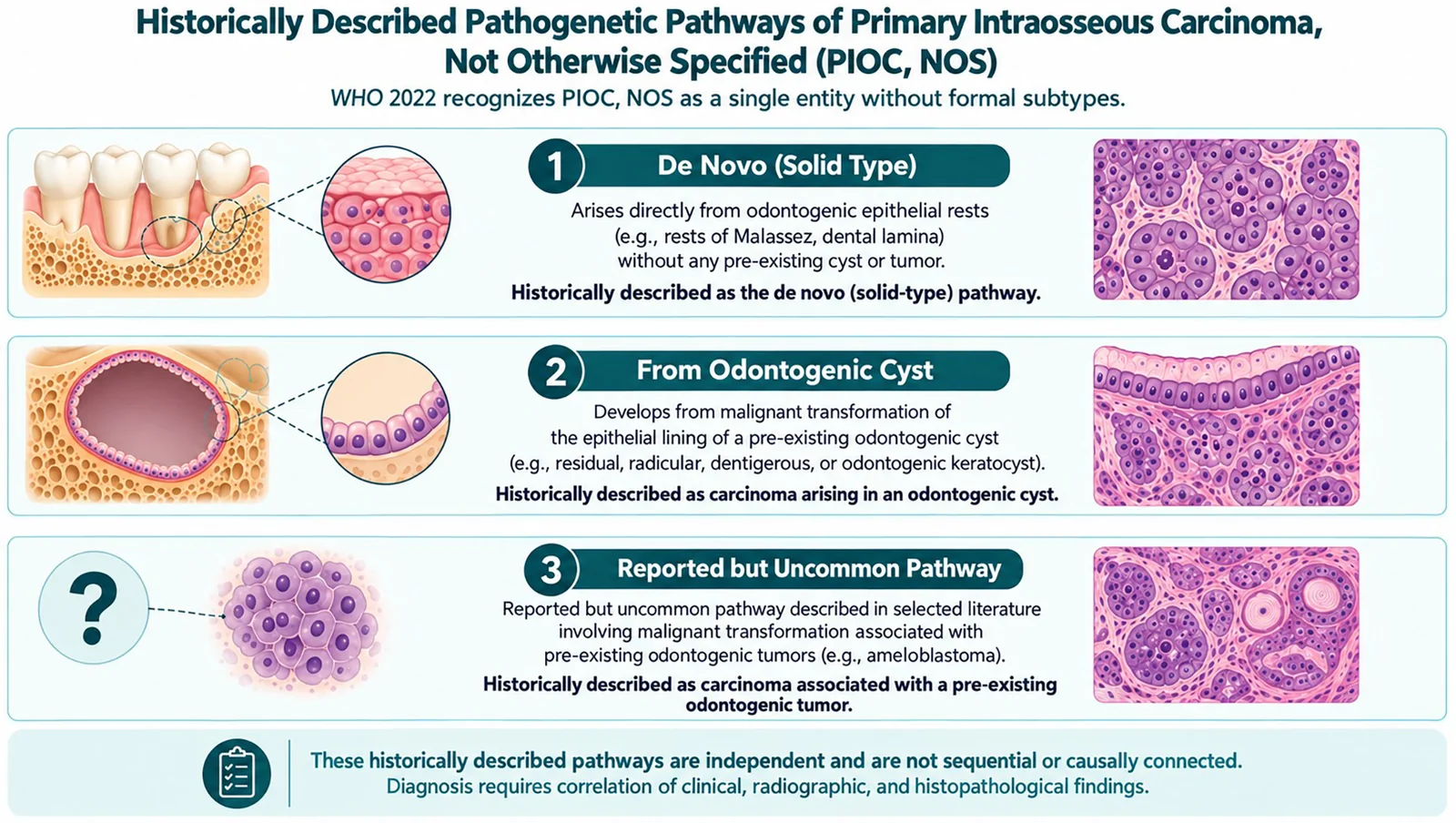
Historically described pathogenetic pathways of PIOC, NOS. Schematic diagram prepared by the authors with the assistance of generative AI (see Generative AI statement), illustrating three developmental pathways historically described in the literature (2): (1) *de novo* (solid type), arising directly from odontogenic epithelial rests such as the rests of Malassez or dental lamina; (2) carcinoma arising from an odontogenic cyst, such as a residual, radicular, dentigerous, or odontogenic keratocyst; and (3) carcinoma associated with a pre-existing odontogenic tumor, for example ameloblastoma. These historical developmental concepts are presented independently and are not sequential or causally connected. The figure is a schematic representation and does not depict patient material. The WHO 2017 and 2022 classifications recognize PIOC, NOS as a single entity without formal subtypes (3, 4).

Histopathological criteria remain the foundation of diagnosis. Suei et al. proposed that diagnosis requires absence of an ulcer of the overlying oral mucosa, absence of another primary tumor at diagnosis and during at least 6 months of follow-up, and histological evidence of squamous cell carcinoma (12). Subsequent clinicopathological studies and reviews have reinforced the importance of keratinization, cellular atypia, infiltrative epithelial nests and bone invasion as key microscopic features (9, 11). These criteria are directly applicable to the present case, which demonstrated well-differentiated squamous epithelial islands with keratinization, nuclear pleomorphism and bone trabeculae partially encased by malignant epithelial nests.

The literature also highlights the aggressive potential of PIOC despite its frequently well-differentiated microscopic appearance. Published clinical series have identified histological grade, nodal involvement or N classification, and treatment modality as important prognostic factors, while postoperative radiotherapy has also been associated with improved overall survival in selected cohorts (9, 13, 14). Five-year overall survival in these series remained approximately 36%–39%, supporting early biopsy, thorough staging and definitive oncologic management. Earlier series and case reports likewise stressed the value of accumulating well-documented cases to improve diagnostic accuracy, facilitate earlier recognition and refine understanding of prognostic factors for this uncommon malignancy (10, 16, 17). Accordingly, this case contributes to the limited literature on solid-type PIOC, NOS by illustrating the cost of diagnostic delay: initial management as an odontogenic infection preceded the diagnosis of a tumor that ultimately measured 7 cm and had metastasized to the cervical lymph nodes. Early correlation of clinical, radiographic and histopathological findings, and prompt biopsy of a destructive lesion that fails to respond to treatment, are therefore essential.

### 3.2 Clinicopathological correlation and key features

Three aspects of this case merit emphasis: the central role of histopathology, the solid (*de novo*) developmental pathway, and the implications for management.

#### 3.2.1 Central role of histopathology

Histopathological evaluation remains the cornerstone of diagnosis. Characteristic findings include infiltrative nests and islands of well-differentiated squamous epithelial cells with nuclear pleomorphism, hyperchromatism, mitotic activity and keratin pearl formation. The presence of bone trabeculae encased by tumor nests, as observed here, confirms intraosseous invasion; when interpreted together with the intact mucosa at presentation and the absence of continuity with surface epithelium, it supports a primary intraosseous origin rather than a surface-derived carcinoma (11, 12). These features, together with the intact mucosa at presentation and the negative staging work-up, satisfy the diagnostic criteria of Suei et al. (12). A focused comparison of the key differential diagnoses is presented in Table 1.

#### 3.2.2 Intact mucosa at presentation and later surface involvement

The resection specimen showed tumor involving the gingiva, gingivobuccal fold and overlying skin, which could be read as evidence against a primary intraosseous origin. However, at first presentation the overlying oral mucosa was clinically intact, without ulceration, surface growth or an intra-oral sinus tract, and the incisional biopsy obtained from within the intraosseous compartment showed no continuity between the tumor and the surface epithelium (Figure 2A). The later mucosal and cutaneous involvement is consistent with secondary extension of the central tumor after the initial presentation. Potential routes include extension through areas of cortical disruption, the extraction or biopsy sites, and the pre-existing cutaneous fistula tract; however, the precise route of extension cannot be determined from the available records. Secondary surface involvement of this kind is recognized in advanced intraosseous carcinoma; the diagnostic criteria refer to the status of the mucosa at initial presentation, which in this case was intact (11, 12).

#### 3.2.3 Pathogenesis and the solid (*de novo*) pathway

The pathogenesis of PIOC is attributed to malignant transformation of odontogenic epithelial remnants, such as the rests of Malassez or cystic linings (2, 3). Although the WHO 2022 classification recognizes PIOC, NOS as a single entity without formal subtypes (3, 4), three presumed developmental pathways have historically been distinguished: carcinoma arising *de novo* (solid type), carcinoma arising in an odontogenic cyst, and carcinoma associated with another odontogenic tumor (2). The present case corresponds to the solid (*de novo*) type, as no pre-existing cyst or tumor was found. Although immunohistochemistry was not performed, CK5, CK14, CK19, p63 and p53 have been evaluated in primary intraosseous squamous carcinoma arising in an odontogenic keratocyst and may provide additional immunophenotypic characterization in diagnostically challenging lesions (18). In addition, next-generation sequencing of two PIOC, NOS cases identified TP53, KDR and APC alterations in one case and no detected mutations in the other, suggesting potential molecular heterogeneity (19). Incorporating such profiling in future cases may further refine diagnostic characterization and prognostic assessment.

#### 3.2.4 Behavior, prognosis and management

Despite its well-differentiated histology, PIOC behaves aggressively, and regional nodal metastasis has been documented in clinical series (9, 13). Because PIOC, NOS arises within bone and has no mucosal surface of origin, depth of invasion — the parameter used for T-classification of mucosal oral squamous cell carcinoma — offers no reference plane from which it could be measured and is not applicable to this tumor; it was not assessed in the present case. Local invasiveness in intraosseous carcinoma is instead judged by markers that reflect intramedullary and extraosseous spread. Radiographically these comprise the maximum tumor dimension and the mesiodistal and craniocaudal extent of medullary involvement on CBCT or CT; the presence, site and completeness of cortical thinning, erosion or frank perforation; extension beyond the periosteum into adjacent soft tissue, including the development of a cutaneous or intra-oral fistula; and involvement of the mandibular canal and inferior alveolar nerve. Histopathologically they comprise the character of the tumor–bone interface (permeative rather than pushing), tumor nests encasing residual bone trabeculae, the presence of perineural or lymphovascular invasion, and, on the resection specimen, surgical margin status and, when available, margin distance. Published series have identified histological grade, nodal involvement or N classification, and treatment modality as important prognostic factors; radiotherapy was also independently associated with overall survival in one institutional cohort (9, 13, 14). Five-year overall survival in these series was approximately 36%–39% (9, 13, 14). In the present case, buccolingual expansion with marked cortical thinning and probable lingual perforation, an associated cutaneous fistula, and the histological demonstration of bone trabeculae encased by malignant epithelial nests together documented local invasiveness without recourse to a depth-of-invasion measurement. Management is primarily surgical: complete resection with adequate oncologic margins, frequently combined with neck dissection because of the risk of cervical metastasis, with adjuvant radiotherapy considered according to adverse pathological features and nodal status (8, 14, 15, 20). Long et al. reported improved overall survival among patients treated with surgery combined with adjuvant therapy and a positive association between radiotherapy and overall survival (14). The treatment adopted in the present case, segmental mandibulectomy with right neck dissection followed by postoperative chemoradiotherapy in view of the nodal metastasis and perineural and lymphovascular invasion, is consistent with this multimodality approach.

Overall, this case underscores the diagnostic value of correlating clinical, radiographic and histopathological findings. Recognition of the hallmark microscopic features — keratin pearls, epithelial invasion of bone and absence of mucosal continuity — is vital to differentiate PIOC from metastatic or surface-derived squamous cell carcinoma and from other odontogenic carcinomas. Early biopsy and heightened clinical awareness remain essential for improving diagnostic accuracy and patient outcomes. This report was prepared in accordance with the CARE (Case Report) guidelines for transparent reporting of clinical case reports; CARE checklist mapping is provided in Supplementary Table S2.

### 3.3 Strengths and limitations

The principal strength of this report is the integrated correlation of clinical, radiographic and histopathological assessment, which together established the diagnosis of solid-type PIOC, NOS while the overlying mucosa was still intact, although the tumor was already locally advanced, supplemented by documentation of the subsequent surgical pathology, postoperative treatment, and oncologic follow-up through September 2026, and the documentation of the case against the CARE checklist. Several limitations must nonetheless be acknowledged. First, immunohistochemical and molecular studies were not performed. Although the morphological criteria and WHO 2022 definitional features were met, additional immunophenotypic characterization using markers such as CK5, CK14, CK19, p63 and p53 could have provided supplementary diagnostic information (18), while molecular profiling might have further characterized tumor heterogeneity (19). Second, although the definitive resection specimen provided additional pathological information, including tumor size, margin status, perineural and lymphovascular invasion, and nodal involvement, detailed operative records were not available to the reporting team. Third, the new mass detected in the right mandibular operative bed in August 2026 was radiologically suspicious for locoregional recurrence, but histopathological confirmation of recurrence was not available. Longer-term outcome and response data following initiation of systemic chemotherapy in September 2026 were also unavailable at the time of reporting. These limitations notwithstanding, the case documents a pattern that is easily missed: an intact mucosa is not a reassuring finding in a destructive central jaw lesion, and treating it as one is the mechanism by which diagnosis is delayed.

## 4 Conclusions

When a destructive central jaw lesion presents beneath an intact overlying mucosa, PIOC, NOS must remain in the differential diagnosis, and the diagnosis ultimately depends on identifying keratin pearls, infiltrative epithelial nests and intraosseous bone invasion in the absence of mucosal continuity. Accurate diagnosis and correct WHO 2022 classification are critical for timely definitive oncologic management. The subsequent nodal involvement, perineural and lymphovascular invasion, and radiologically suspected locoregional recurrence in this case further underscore the aggressive behavior of PIOC, NOS and the importance of continued oncologic surveillance after treatment.

## Data Availability

The raw data supporting the conclusions of this article will be made available by the authors, without undue reservation.
