# Supplementary material for: When intact mucosa hides a malignancy: solid-type primary intraosseous carcinoma (PIOC, NOS) of the mandible: a case report and literature review
Source: Front Dent Med. 2026 Oct 14;7:1918737. doi: 10.3389/fdmed.2026.1918737 (PMC13713352; doi:10.3389/fdmed.2026.1918737)
Supplement: Supplementary file 1 [file Table1.docx]

**Table S1: Timeline of clinical events, diagnostic assessment, intervention, and follow-up**

**A chronological summary of the clinical course, diagnostic assessment, treatment, and follow-up is presented in Table S1.**

| **Date / time point** | **Clinical event / investigation / intervention** |
| --- | --- |
| Initial clinical course (cutaneous fistula reported for approximately 1 month) | Painful swelling of the right mandibular/cheek region associated with purulent discharge through a cutaneous fistula. The condition was initially considered an odontogenic abscess and was treated with antibiotics and local drainage; symptoms persisted. |
| 30 Aug 2025 | CBCT demonstrated a destructive intraosseous lesion involving the right posterior mandible in the first-to-third molar region, with loss of normal trabecular architecture, buccolingual expansion, marked cortical thinning, and probable focal cortical discontinuity/perforation. |
| Between 30 Aug and 7 Sep 2025; exact date unavailable | Following CBCT, the right mandibular first and third molars, located within the affected region, were extracted. Abnormal, friable-appearing tissue was encountered at the extraction sites, prompting histopathological investigation. |
| 7 Sep 2025 | An incisional biopsy was obtained through the extraction sockets from abnormal tissue within the intraosseous lesion; the specimen comprised tumor tissue with fragments of residual trabecular bone. |
| 10 Sep 2025 | Histopathological examination established the diagnosis of primary intraosseous carcinoma, not otherwise specified (PIOC, NOS), under WHO 2022; the tumor was well differentiated (Grade I) and corresponded to the historically described solid (de novo) type. |
| 17 Sep 2025 | Staging contrast-enhanced CT of the brain, neck, and chest identified no suspicious primary tumor within the imaged regions. |
| 1 Oct 2025 | Definitive oncologic surgery was performed, comprising segmental mandibulectomy of the right mandible with right neck dissection. |
| Oct 2025 (after decalcification of the resection specimen) | Final surgical pathology showed a 7-cm invasive well-differentiated keratinizing squamous cell carcinoma involving the gingiva and gingivobuccal fold, underlying soft tissue, mandibular bone, and overlying skin with fistula-tract formation. Perineural and lymphovascular invasion were identified. Four level I lymph nodes, submitted with the attached submandibular gland, showed metastatic tumor; the remaining 12 right-neck lymph nodes were reactive (overall, 4/16 lymph nodes involved). All surgical margins were free of tumor. |
| 30 Oct 2025 | Postoperative CT demonstrated postsurgical changes in the right mandibular operative bed. |
| 9 Dec 2025 | Postoperative radiotherapy commenced at 200 cGy (2 Gy) per fraction for 20 fractions, for a total dose of 4000 cGy (40 Gy); the course was extended by treatment interruptions. |
| 14 Jan 2026 | Cisplatin 50 mg was administered concurrently during the postoperative radiotherapy course. |
| 12 Aug 2026 | Follow-up contrast-enhanced CT demonstrated a new heterogeneous soft-tissue mass in the right mandibular operative bed, measuring approximately 4.5 × 3.9 × 4.0 cm, radiologically suspicious for locoregional recurrence. |
| 27 Aug 2026 | Comparative radiologic review of the 12 Aug 2026 CT against the postoperative CT of 30 Oct 2025 confirmed the new heterogeneous mass in the surgical bed; clinical and histopathological correlation was recommended. |
| 21 Sep 2026 | Systemic chemotherapy with paclitaxel (250 mg) and carboplatin (450 mg) was initiated. |
| Status at last follow-up (Sep 2026) | Radiologically suspected locoregional recurrence under systemic chemotherapy; histopathological confirmation of recurrence and treatment-response data were not yet available. |
