# Supplementary material for: When intact mucosa hides a malignancy: solid-type primary intraosseous carcinoma (PIOC, NOS) of the mandible: a case report and literature review
Source: Front Dent Med. 2026 Oct 14;7:1918737. doi: 10.3389/fdmed.2026.1918737 (PMC13713352; doi:10.3389/fdmed.2026.1918737)
Supplement: Supplementary file 2 [file Table2.docx]

**Supplementary Material**

**Table S2: CARE Checklist Mapping**

The table below maps each CARE checklist item to the corresponding section of the manuscript, in accordance with the 2013 consensus statement and the 2017 explanation and elaboration document.

| **CARE item** | **Topic** | **Where addressed** |
| --- | --- | --- |
| 1 | Title — diagnosis and “case report” identified | Title page |
| 2 | Key words | Abstract (end) |
| 3 | Abstract (structured) — background, case, conclusions | Abstract |
| 4 | Introduction — background, literature context | Section 1 |
| 5a | Patient information — demographics, history, relevant comorbidities | Section 2.1 |
| 5b | Main complaint and presenting concern | Section 2.1 |
| 6 | Clinical findings — relevant physical and clinical examination findings | Section 2.2 |
| 7 | Timeline — historical and current information in chronological order | Section 2.3 and Table S1 |
| 8a | Diagnostic assessment — diagnostic methods, challenges, reasoning | Sections 2.4.1–2.4.5 |
| 8b | Diagnostic reasoning including differential diagnosis | Sections 2.4.4–2.4.5 and Table 1 |
| 9a | Therapeutic intervention — types and details | Section 2.5 |
| 9b | Changes in intervention with rationale | Sections 2.5–2.6 (postoperative chemoradiotherapy and subsequent systemic chemotherapy after radiologically suspected recurrence) |
| 10 | Follow-up and outcomes — clinician- and patient-assessed outcomes, adverse events | Section 2.6 (treatment-response data beyond initiation of systemic chemotherapy were not available at last follow-up) |
| 11 | Discussion — strengths, limitations, comparison with literature, take-away lessons | Sections 3.1–3.3 and Section 4 |
| 12 | Patient perspective | Not reported / not available |
| 13 | Informed consent | Ethics statement (Declarations) |

**Table S2.** Mapping of CARE checklist items to manuscript sections.
